# First-Trimester Radical Hysterectomy for Bulky Cervical Cancer: A Case Report on Oncologic and Reproductive Considerations

**DOI:** 10.7759/cureus.82486

**Published:** 2025-04-18

**Authors:** Munachiso I Ndukwe, Denisa Pohankova, Petr Halada, Jan Laco, Martin Stepan, Tatana Reslova, Petra Bretova, Dominik Karasek, Domink Habes, Adam Rezac, Igor Sirak

**Affiliations:** 1 Department of Obstetrics and Gynaecology, University Hospital Hradec Kralove, Charles University, Faculty of Medicine, Hradec Kralove, CZE; 2 Department of Oncology and Radiotherapy, University Hospital Hradec Kralove, Charles University, Faculty of Medicine, Hradec Králove, CZE; 3 Department of Oncology and Radiotherapy, University Hospital Hradec Kralove, Charles University, Faculty of Medicine, Hradec Kralove, CZE; 4 The Fingerland Department of Pathology, University Hospital Hradec Kralove, Charles University, Faculty of Medicine, Hradec Kralove, CZE

**Keywords:** open radical hysterectomy, pregnancy, surgical abortion, uterine cervical cancer, women's reproductive rights

## Abstract

The management of cervical cancer during the first trimester of pregnancy requires a multidisciplinary approach that considers medical, psychological, and social factors. This report details the case of a patient diagnosed preoperatively with bulky early-stage cervical cancer at nine weeks of gestation, during an evaluation initially intended for pregnancy termination. While definitive chemoradiotherapy is the standard treatment for early-stage bulky cervical tumors, the treatment strategy, in this case, was influenced by the patient’s decision to terminate the pregnancy without delay. A radical hysterectomy with pelvic lymphadenectomy was successfully performed at 10 weeks of gestation. The patient experienced an uneventful postoperative recovery and was subsequently advised to undergo adjuvant chemoradiotherapy. This case underscores the importance of an individualized management plan that prioritizes maternal health while respecting reproductive rights and patient autonomy.

## Introduction

Cervical cancer remains the fourth most prevalent malignancy among women worldwide, accounting for a significant proportion of cancer-related morbidity and mortality [1]. Its incidence has shown a substantial decline in developed nations, largely attributed to the widespread adoption of human papillomavirus (HPV) vaccination programs and routine cervical dysplasia screening through Pap smears and HPV testing [2]. In pregnant women, cervical cancer is rare, particularly in developed nations, and is usually diagnosed in the early stages during the first-trimester cervical screening [3]. Management during pregnancy poses unique challenges, particularly in the first trimester, when therapeutic options are limited. Both chemotherapy and radiotherapy are contraindicated during this period due to their teratogenic effects, making surgery the preferred and often sole treatment modality [4]. Effective management requires a multidisciplinary approach, considering the disease stage, the patient's preferences regarding pregnancy outcomes, and the gestational age [5].

This case report presents the clinical course of a patient with an apparent bulky early-stage cervical cancer, diagnosed in the first trimester of pregnancy. Notably, this is the first case report in the literature in which a pregnant patient with cervical cancer expressed a desire for a medical abortion due to strong personal reasons, which influenced the treatment decisions. The report discusses the therapeutic decision-making process, maternal and fetal outcomes, and the implications of such interventions for pregnant women with early-stage cervical cancer.

## Case presentation

Patient information

**Figure 1:**
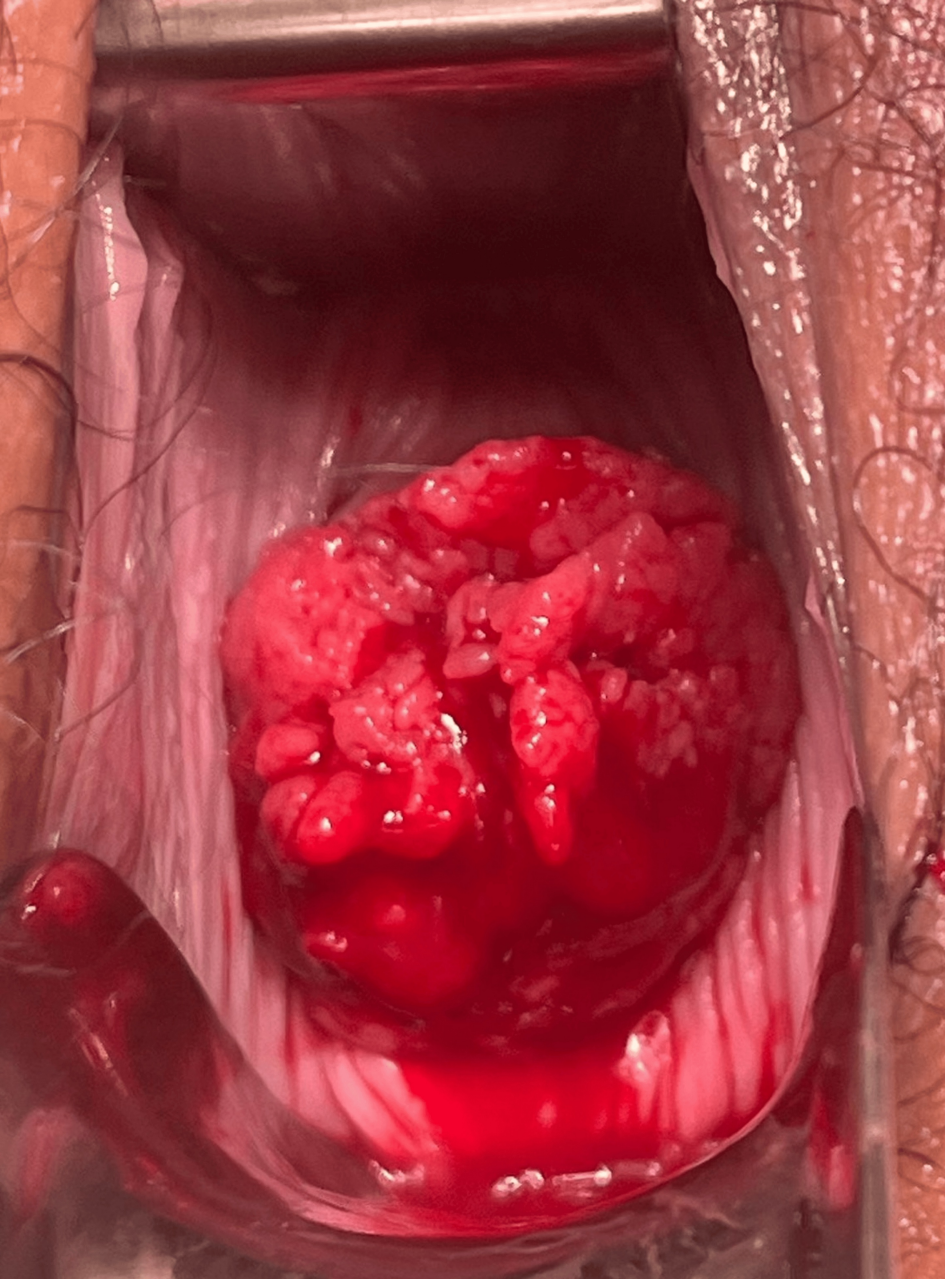
Speculum examination before the planned abortion in the first trimester

A 32-year-old woman (gravida 4, para 3) at nine weeks of gestation was referred to a private clinic for pregnancy termination due to an unwanted pregnancy. This referral followed an ultrasound evaluation by her outpatient gynecologist. However, during the pre-abortion examination at the private clinic, the gynecologist raised suspicion of cervical cancer (Figure 1). A punch biopsy was performed and sent to our expert pathologist in gynecological oncology at University Hospital Hradec Králové, leading to the deferral of the abortion plan. The patient was subsequently referred to our specialized gynecologic oncology center for further management on the same day. She had no significant medical or surgical history and had a body mass index (BMI) of 21.9.

Diagnosis and imaging

Upon admission, a vaginal examination revealed a bulky tumor with contact bleeding, most likely originating from the cervix. An expedited histological examination conducted by the expert pathologist in gynecological oncology from the punch biopsy confirmed the diagnosis of invasive, p16-positive squamous cell carcinoma of the cervix, with no evidence of lymphovascular or perineural invasion. 

A transrectal and transabdominal ultrasound was performed by a certified gynecological oncology sonographer. The uterus measured 119 × 57 × 73 mm, and the uterine cervix measured 37 × 31 × 36 mm. A single viable intrauterine pregnancy in cephalic presentation was identified, with a crown-rump length of 27.4 mm, corresponding to a gestational age of nine weeks and four days. A cervical tumor measuring 43 x 35 x 45mm was observed arising from the anterior exocervix, with intimate contact to the anterior vaginal fornix and a color Doppler score of 4, indicating increased vascularity. No evidence of invasion into the parametria, urinary bladder, rectum, or adnexa was noted. Transabdominal ultrasound showed no signs of positive pelvic or paraaortic lymph nodes or distant metastases. Based on these findings, a FIGO IB3 cervical tumor was suspected, with a potential extension to FIGO IIA. Magnetic resonance imaging (MRI) of the pelvis, performed without intravenous contrast and interpreted by a radiologist specializing in gynecological oncology, concurred with the ultrasound conclusion. In addition, a chest X-ray was performed, which revealed no abnormalities.

Therapeutic decision

The patient was discussed at a multidisciplinary team meeting attended by a clinical oncologist, radiation oncologist, gynecological oncologist, pathologist, radiologist, and psychologist, where multiple factors were considered in the decision-making process. These included the patient’s expressed desire to terminate the pregnancy for strong personal reasons, the presence of a bulky tumor obstructing access to the external cervical os, favorable histological prognostic factors such as the absence of lymphovascular and perineural invasion, and the lack of clear evidence of invasion beyond the uterine cervix and vagina, as well as no lymphadenopathy on preoperative imaging.

The first two factors were decisive in the decision-making process, as performing a dilation and vacuum aspiration was deemed impossible in this patient. In addition, a pharmacologically induced abortion at 10 weeks of gestation was considered extremely risky. Consequently, the tumor board recommended a nerve-sparing radical hysterectomy with pelvic lymphadenectomy. Sentinel lymph node detection was not indicated intraoperatively, as there was no healthy part of the cervix for indocyanine green injection [6]. Moreover, the radical hysterectomy was to be performed regardless of sentinel lymph node status due to the previously mentioned considerations.

Surgery

**Figure 2:**
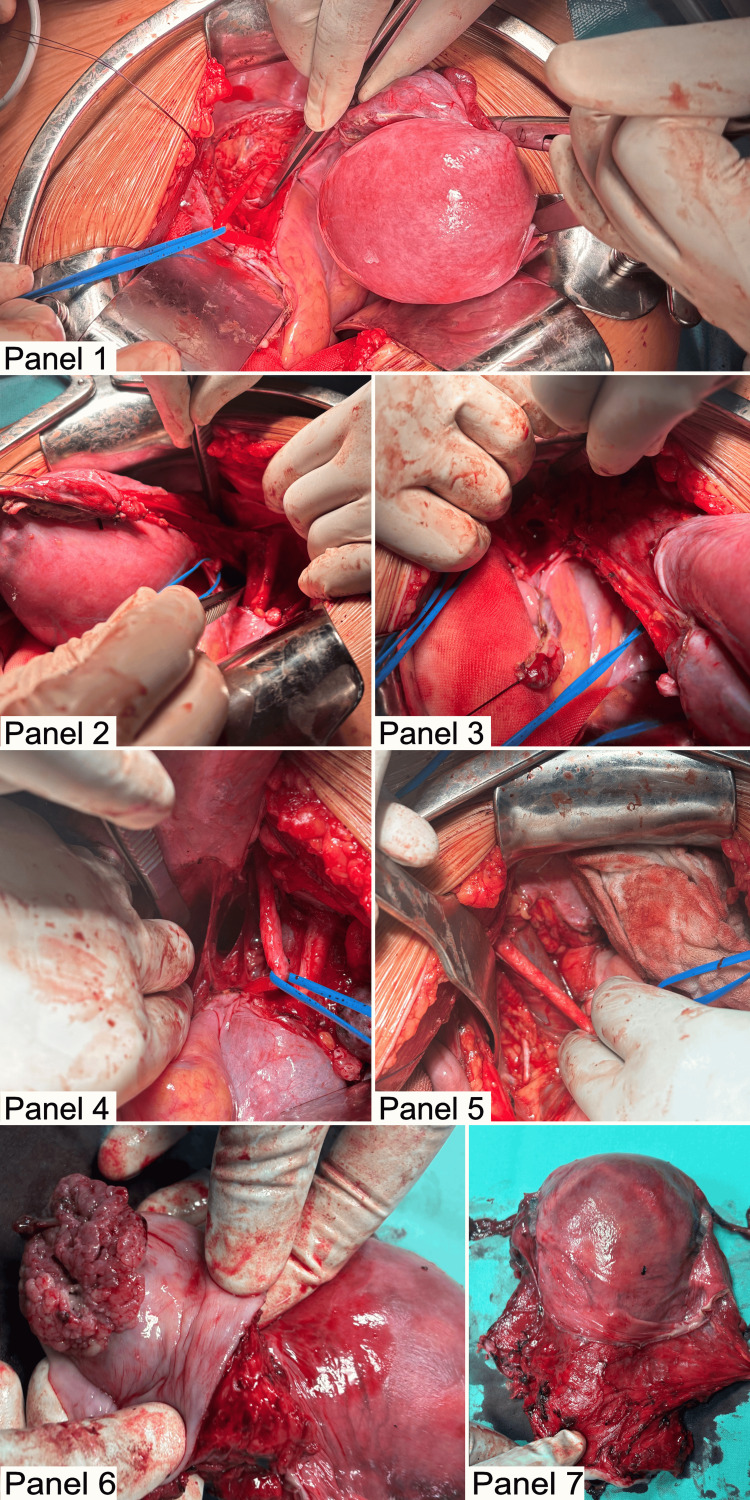
Surgical proceedure with definite specimen after radical hysterectomy Panel 1: Left Okabayashi space lateral to the ureter, Panel 2: Right paravesical and pararectal spaces, Panel 3: Skeletonized suspensory ligament of the left ovary prior to transposition, Panel 4: Right Okabayashi and pararectal spaces with the right ureter lateralized, Panel 5: Left external iliac artery and vein with the obturator nerve exposed, Panel 6: Vaginal cuff following radical hysterectomy, Panel 7: Surgical specimen post radical hysterectomy.

A midline laparotomy was performed, providing complete exploration of the peritoneal cavity and retroperitoneal space. The uterus was noted to be enlarged, with engorged varices identified in the broad ligament; no enlarged lymph nodes were identified. A C1 radical hysterectomy, classified according to the Querleu-Morrow system, was performed [7,8]. This included removal of the uterine body, cervix, upper one-third of the vagina, and wide resection of the parametrium, while preserving the hypogastric nerve. A systematic bilateral pelvic lymphadenectomy was conducted up to the level of the common iliac arteries, along with a bilateral prophylactic salpingectomy and transposition of the ovaries (Figure 2). The procedure was completed without perioperative complications, utilizing a bipolar vessel sealer, with an estimated blood loss of 200 mL. The patient had an uneventful postoperative course and was discharged at her request on postoperative day 4.

Histopathological examination 

Gross Findings

Macroscopic examination revealed a uterus measuring 140 × 90 × 75 mm and weighing 420 g, with bilateral fallopian tubes (left: 75 × 5 × 5 mm; right: 80 × 5 × 5 mm), parametrium (left: 40 × 25 × 25 mm; right: 45 × 35 × 30 mm), and a vaginal cuff of 20 mm.

An exophytic tumor measuring 45 × 35 × 35 mm was observed on the cervix between the 10 and 2 o’clock positions. The tumor did not extend into the uterine body, parametrium, or vaginal cuff. It was firm in consistency and whitish in color on sectioning. The tumor was entirely submitted for microscopic examination. Within the uterine cavity, a single 40-mm fetus in cephalic position was present, with a 40-mm umbilical cord centrally attached to the placenta's chorionic villi, which showed no abnormalities. The myometrium measured 15-20 mm in thickness and exhibited no pathological changes.

Microscopic Findings

Tissue specimens were fixed in formalin and routinely processed for histopathology. Immunohistochemistry for p16 protein expression was performed on 2-μm-thick sections of formalin-fixed, paraffin-embedded tissue using the Omnis immunostainer (Dako/Agilent, Santa Clara, CA, USA) and an anti-p16 antibody (dilution 1:100, clone R15-A, DB Biotech, Kosice, Slovakia), according to the manufacturer’s recommendations.

**Figure 3:**
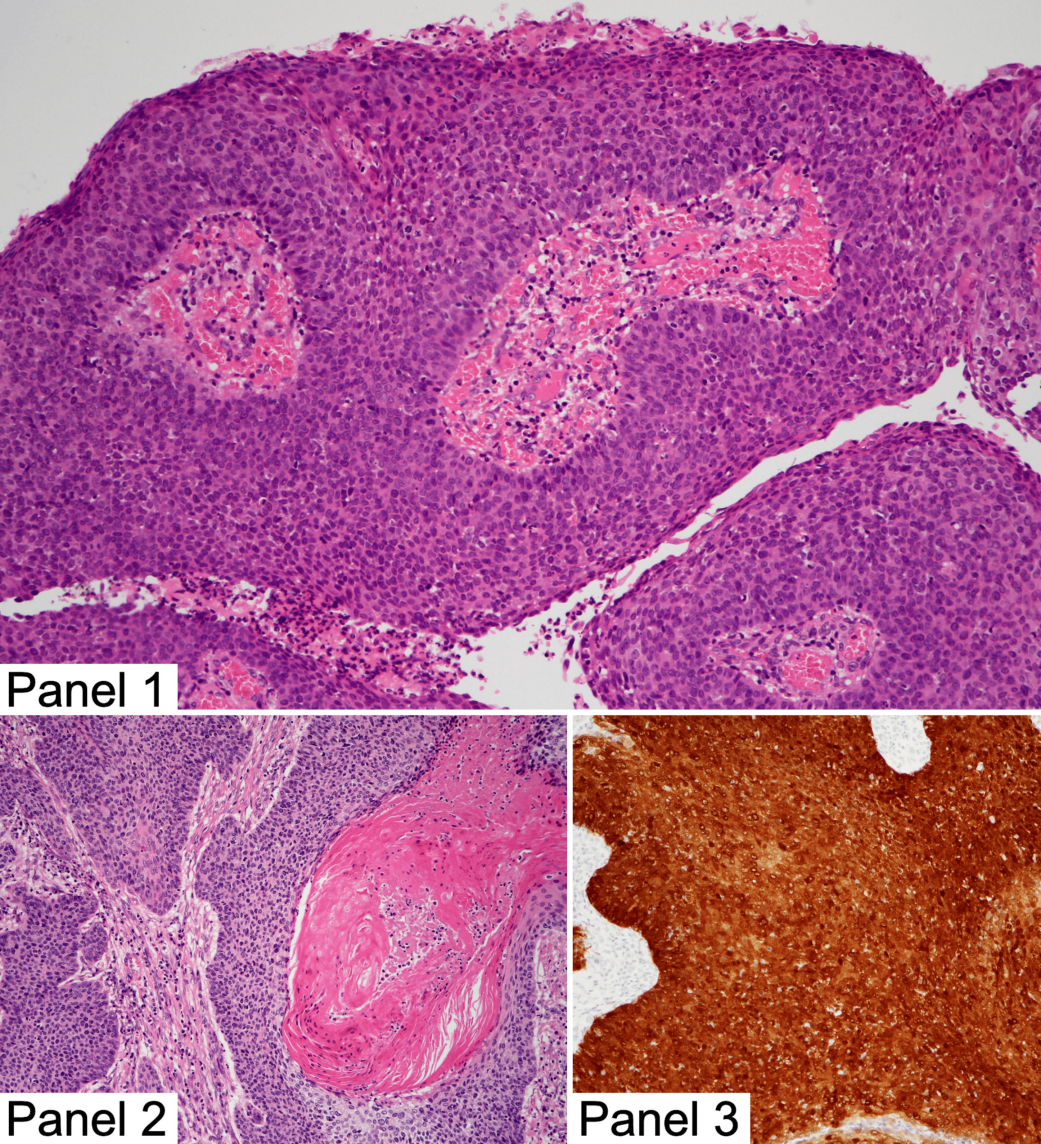
Histopathological image captured by our in-house gynecological oncology specialist. Panel 1: Papillary growth pattern in the carcinoma, Panel 2: Infiltrative invasion in the carcinoma, Panel 3: p16 expression in the carcinoma

Microscopically, the tumor was diagnosed as invasive squamous cell carcinoma of the cervix with focal keratinization (Figure 3). Grading is not defined for this type of cervical malignancy. The carcinoma measured up to 45 mm in greatest dimension. It infiltrated the superficial third of the cervical stroma, with a maximum stromal invasion depth of 11 mm. Lymphovascular invasion was present, but there was no invasion into blood vessels or perineural spread. No microscopic spread was observed to the uterine body, parametrium, or vaginal cuff. Resection margins were negative: ectocervical/vaginal cuff at 7 mm and radial/deep stromal margin at 11 mm. Strong nuclear and cytoplasmic expression of p16 protein was seen in 100% of tumor cells (Figure 3).

Adjacent to the invasive carcinoma, high-grade squamous intraepithelial lesion (HSIL) was identified on the cervix. It did not extend to the vaginal cuff and was located 25 mm from the ectocervical/vaginal cuff resection margin. Of the 24 lymph nodes examined, two from the left obturator fossa were positive-one with a 6.5-mm metastasis and another with a 1.3-mm micrometastasis. No extranodal extension was observed.

The final diagnosis was HPV-associated (p16-positive) squamous cell carcinoma of the cervix (ICD-O 8085/3). The tumor was staged as pT1b3 pN1(24/2) cM0 L1 V0 Pn0 R0 (according to UICC Cervix Uteri TNM 2021) and as FIGO stage IIIC1 (according to FIGO 2018).

Microscopic examination of the fetus revealed no significant abnormalities. The umbilical cord contained three vessels and showed no signs of inflammation. The fetal membranes showed foci of old hemorrhage with mild acute inflammation. The chorionic villi were normal in size and shape and were attached to the decidua.

Follow-Up and Postoperative Care

On the 10th postoperative day, suture removal revealed primary healing of the laparotomy site without signs of infection, dehiscence, or complications. The wound edges were well approximated, and the surrounding tissue appeared healthy. By the 21st postoperative day, a thorough evaluation, including physical examination and ultrasonographic imaging, showed no abnormal findings. The patient reported no significant postoperative symptoms such as pain, fever, or discomfort. Ultrasonography confirmed normal pelvic anatomy with no fluid collection, hematoma, or other complications, consistent with the expected healing trajectory.

The multidisciplinary team recommended a positron emission tomography-computed tomography (PET-CT) scan to rule out positive para-aortic lymph nodes or distant metastases. After a comprehensive discussion with the patient, concomitant chemoradiotherapy was advised, comprising pelvic external beam radiotherapy and brachytherapy in combination with Cisplatin. The patient opted for treatment in a regional center.

## Discussion

Cervical cancer in pregnancy presents a unique challenge, both in terms of clinical management and the ethical considerations surrounding treatment options. While cervical cancer is relatively uncommon during pregnancy, its diagnosis requires prompt and careful decision-making to balance maternal survival and fetal health. 

The treatment algorithm for cervical cancer in early pregnancy can be categorized into two approaches: pregnancy preservation or pregnancy termination. During the first trimester, pregnancy preservation options include conization, simple trachelectomy, or radical trachelectomy, with or without lymph node staging, depending on tumor size and lymphovascular invasion [9]. Sentinel node biopsy has been demonstrated as a feasible option for pregnant women with early-stage cervical cancer [10]; however, it is still considered an experimental method according to current European guidelines [11]. Another approach for pregnancy preservation in the first trimester involves delaying treatment until after the 13th week to administer neoadjuvant chemotherapy, followed by radical trachelectomy [12]. In cases where the patient opts for pregnancy termination, management should follow established guidelines, prioritizing maternal health while tailoring the care plan to the patient’s specific clinical presentation.

## Conclusions

This case report underscores the critical need for an individualized, multidisciplinary approach in the management of cervical cancer during pregnancy. It highlights the importance of integrating medical, psychological, and social considerations to optimize patient outcomes. Furthermore, it illustrates the complex challenges inherent in balancing oncological treatment with the unique physiological and ethical considerations of pregnancy, reinforcing the necessity of tailored decision-making in such cases.
